# Venous Thromboembolism in Cancer Patients on Simultaneous and Palliative Care

**DOI:** 10.3390/cancers12051167

**Published:** 2020-05-06

**Authors:** Silvia Riondino, Patrizia Ferroni, Girolamo Del Monte, Vincenzo Formica, Fiorella Guadagni, Mario Roselli

**Affiliations:** 1 Department of Systems Medicine, Medical Oncology, University of Rome, Tor Vergata, 00133 Rome, Italy; silviariondino2@gmail.com (S.R.); vincenzo.formica@ptvonline.it (V.F.); 2 Department of Human Sciences and Quality of Life Promotion, San Raffaele Roma Open University, 00166 Rome, Italy; patrizia.ferroni@sanraffaele.it (P.F.); fiorella.guadagni@sanraffaele.it (F.G.); 3 Department of Palliative Care, San Raffaele Cassino, Clinical Center, 03043 Cassino, Italy; girolamo.delmonte@sanraffaele.it

**Keywords:** simultaneous care, integrated care, venous thromboembolism, thromboprophylaxis

## Abstract

Simultaneous care represents the ideal integration between early supportive and palliative care in cancer patients under active antineoplastic treatment. Cancer patients require a composite clinical, social and psychological management that can be effective only if care continuity from hospital to home is guaranteed and if such a care takes place early in the course of the disease, combining standard oncology care and palliative care. In these settings, venous thromboembolism (VTE) represents a difficult medical challenge, for the requirement of acute treatments and for the strong impact on anticancer therapies that might be delayed or, even, totally discontinued. Moreover, cancer patients not only display high rates of VTE occurrence/recurrence but are also more prone to bleeding and this forces clinicians to optimize treatment strategies, balancing between hemorrhages and thrombus formation. VTE prevention is, therefore, regarded as a double-edged sword. Indeed, while on one hand the appropriate use of antithrombotic agents can reduce VTE occurrence, on the other it significantly increases the bleeding risk, especially in the frail patients who present with multiple co-morbidities and poly-therapy that can interact with anticoagulant drugs. For these reasons, thromboprophylaxis should start while active cancer treatment is ongoing, according to a simultaneous care model in a patient-centered perspective.

## 1. Integrated Palliative Care and Simultaneous Care

In the new era of personalized medicine, one of the recognized priorities regards the role of integration of early supportive and palliative care with cancer-directed treatments, the so-called “simultaneous care” [1]. It has been widely acknowledged that such an integrated model of care may have a positive effect on patients’ quality of life (QoL) as well as on other patient outcomes [2].

The very notion of “simultaneous care” has stem from the necessity of an earlier integration between oncology and palliative care and from the observation that earlier interventions resulted in reduced depression and symptom burden, decreased hospital care, improved QoL and prolonged survival [3,4,5]. This concept has been implemented by the World Health Organization (WHO), stating that palliative care is already applicable early in the course of illness, in conjunction with other therapies that are intended to prolong life [6]. 

The concept of palliative care has thus substantially developed from its initial meaning of care for the dying, towards a more complete, yet complex, integrated approach focused on patient centered care. Indeed, the WHO describes “palliative care” as an approach that improves the quality of life of patients and their families facing the problems associated with life-threatening illness, through the prevention and relief of suffering by means of early identification and impeccable assessment and treatment of pain and other distresses, at physical, psychosocial and spiritual level [6]. 

Although historically supportive care has been developed to counter and mitigate the side effects of cancer treatment, such as chemotherapy-induced nausea and vomiting (CINV) or neutropenia, while palliative care is intended when the patient is “out of therapy,” when active cancer treatments are no longer available, symptom management is the shared goal of both care settings. The European Society of Medical Oncology (ESMO) also encourages the role of supportive care at all stages of the disease and considers palliative care focused on treatments that take place when active anticancer therapies are no more indicated [7]. The transition between simultaneous and palliative care should be taken following the evaluation of some “guiding” indexes, including disease progression, worsening of performance and/or nutritional status, weight loss and symptom burden [8,9]. The integration of oncology treatment and palliative care is steered by a patient’s centered approach and, by virtue of this integration, treatment outcome must be continuously evaluated and redefined during the progression of the disease and must always be aimed at controlling symptoms and at maintaining the longest possible well-being [8], in order to ensure compliance to a congruent treatment.

In this light, to the National Comprehensive Cancer Network (NCCN) guidelines, patients should be screened at every visit, starting from the very beginning and during follow-ups and checked for the following items—1) uncontrolled symptoms; 2) moderate to severe distress related to cancer diagnosis and therapy; 3) serious comorbid physical, psychiatric and psychosocial conditions; 4) life expectancy of 6 months or less; 5) patient or family concerns about the disease course and decision-making; and/or 6) a specific request for palliative care by the patient or family [10]. 

Accordingly, three different scenarios can be delineated, in which cancer patients can fluidly move during the course of their disease—primary, secondary and tertiary palliative care [11]. Although all settings require professional skills aimed at performing basic assessments and management of physical symptoms, including the evaluation of the drug-induced ones, socio-psychological problems and caregiver support, primary palliative care can occur both as outpatient setting for ambulatory patients and in the home setting, secondary palliative cares are provided in specialized cancer centers at inpatient- and outpatient- levels and tertiary palliative care requires the presence of palliative care specialists [11]. In the perspective of changes, the simultaneous care also overcomes the drawback relative to life expectancy of 6 months or less [10] which, unlike what has been implied so far in the concept of palliative care, is no longer considered in a temporal viewpoint but in a symptomatic one. 

The changes introduced in the new conception of oncology care have thus led to include not only active anticancer under this model but also strategies aimed at primary prevention, early diagnosis, cure, survival-prolongation, supportive treatment and tertiary prevention, including rehabilitation that, together with continuous care and palliative care, extends during the whole course of the disease and even beyond. Indeed, in cancer survivals, physical symptoms and psychological distress altering and affecting their QoL, continuous care should be provided, being capable to improve the perceptions of their lives [12]. Current guidelines recommend the early integration of PC for patients with cancer [3,13], due to the demonstrated positive relationship between early integration of PC and improvements in QoL of both patients and caregivers, associated with a reduced access to pointless interventions and incongruent emergency department accesses [14,15,16,17]. 

Despite these considerations and recommendations, cancer patients are often referred to PC late in their disease course. Towards the final phases of the disease, home-based care is particularly important, because it can prevent inadequate hospital admissions and allows patients to live in their own environment where, however, they must be dynamically evaluated in order to identify their needs that change over time in response to treatment or disease progression. Patients’ needs must be always used in the decision making process and discussed with the patients themselves, in a patient-centered approach [11]. Patient “centeredness” has been defined as “care that is respectful of and responsive to, individual patient preferences, needs and values and ensuring that patient values guide all clinical decisions” [18]. In this light, the aim of patient-centeredness is to personalize treatment and care to the specific needs of each patient and to modulate the care accordingly. 

## 2. Incidence of VTE in Palliative Cancer Patients—A Road Map Approaching Simultaneous Care

Starting from 1995, the National Council for Hospice and Specialist Palliative Care Services has set a systematic data collection of minimum standard (The Minimum Data Set (MDS)) to inform and analyze the activity of Palliative Care Services. MDS methods have been adopted worldwide by most of Palliative Care Organizations and have allowed consistent data comparison, including venous thromboembolism (VTE) in specialist palliative care units (SPCUs) [19].

VTE in its two tightly related clinical entities, deep vein thrombosis (DVT) and pulmonary embolism (PE) [20], is commonly considered as one of the preventable sequelae that can afflict hospitalized patients. In cancer patients VTE occurs in approximately 20% cases [21] and PE is associated with a mortality rate up to 30% [22,23]. Although representing the extreme expression of a manifest disease, asymptomatic manifestations, including disseminated intravascular coagulation, occur rather frequently and are often detected at time of restaging.

VTE incidence increases with age, represents a frequent complication of the more advanced stages and is significantly worsened by anticancer treatments such as platinum-, fluoropyrimidine- and gemcitabine-containing chemotherapy regimens, immune-modulatory drugs such as thalidomide, lenalidomide and pomalidomide, estrogen receptor inhibitor tamoxifen, antiangiogenic drugs, administered both alone and in combination and by concomitant supportive therapies (i.e., granulocyte-colony stimulating factors, erythropoiesis stimulating agents and glucocorticoids) [24,25,26,27,28]. The highest association with VTE is observed for multiple clinical factors, including increased medical co-morbidities and treatment associated with multiple drugs. The association between VTE and cancer appears to be time-dependent, with most VTE events occurring within the first 6 months after cancer diagnosis and the first 3 months of chemotherapy [25,29,30]. If on one hand surgery and medical treatment, catheters, chemo-radiotherapy and co-morbidities contribute to increase thrombosis risk [31], on the other some co-morbidities and/or chemotherapy-related side effects, such as renal or hepatic insufficiency and thrombocytopenia, can affect the efficacy and safety of anticoagulation [32].

These factors associated with age and ECOG (Eastern Cooperative Oncology Group) performance status, indicative of the patient’s level of function and mobility [33,34] are parameters that drive all decision making processes in cancer patients at risk for VTE and which should indicate not only those who might benefit from thromboprophylaxis but also those who are fit for receiving it. Indeed, the increased risk of VTE occurrence and/or recurrence requires a tight balance with the anticoagulation-associated major bleeding complications [35,36,37]. The interplay between VTE and cancer has been further confirmed by considering that ~20% of patients with an episode of unprovoked VTE will be diagnosed with cancer within one year from VTE occurrence [38] and that patients with malignancies are more prone than others to develop recurrent VTE despite appropriate anticoagulant therapy [29,35,37].

In centers adhering to MDS standards, admission to Nursing Homes with a diagnosis of symptomatic VTE is reported to be overall (independently of cancer diagnosis) ~4% [39], while ~1% of new symptomatic VTE are recorded during residency [40]. These figures significantly increase for SPCUs dealing with advanced cancer patients, with a recorded ~10% prevalence [41]. 

In a study by Soto-Cárdenas et al., 712 patients attending the SPCU in a three-year period of observation presented a symptomatic VTE in 9.98% of cases (*n* = 71) [42]. Lung and colorectal cancer were most prevalent primary tumors (14% and 13%, respectively) and for the vast majority of patients (88.7%) symptomatic VTE occurred in the outpatient setting and was itself the cause of admission to SPCU.

Two studies have also investigated the incidence of asymptomatic VTE in these sceneries. Johnson et al. have analyzed the presence of cloths in deep veins (DVT) of 258 hospice cancer inpatients by means of light reflection rheography. The prevalence of DVT was 52% (*n* = 135) and being bedridden and hypoalbuminemia were independent risk factors at multivariate analysis [43].

In a prospective longitudinal trial, the HIDDen trial, 273 SPCU cancer patients from the UK, with a life expectancy >5 days, were screened for asymptomatic DVT by using bilateral femoral vein ultrasonography within 48 h of admission [44]. Stringent follow-up was also carried out with weekly repeated ultrasonography up to 3 weeks after admission. As expected, study population was relatively elderly (mean age 68 years) and with a poor Karnofsky performance status (mean score: 49).

Consistent results with Johnson’s study were demonstrated, with an incidence of DVT of 34% and being bedbound in the 12 weeks preceding study recruitment as a significant risk factor [44]. 

Albuminemia was not confirmed to be associated with DVT diagnosis (*p* = 0.43). Interestingly, only four additional patients were diagnosed with DVT during the three weeks of follow-up.

Overall, these data confirmed that the prevalence of VTE among palliative care unit (PCU) patients is impressive (35–50%), however how much it might truly affect QoL in this cancer setting is still highly debated, although in terms of increasing anxiety, patients consider VTE a physically and emotionally distressing phenomenon that overlaps the underlying malignancy and strongly decreases their QoL. The PELICAN study [45], performed by interviewing a small population of cancer patients in order to assess their perception of the newly diagnosis of cancer-associated thrombosis (CAT), has identified three stages that were described as “life before CAT,” “initial diagnosis and treatment of CAT” and “living with CAT,” each one associated with specific patient needs. The study showed that only an exhaustive information by the clinical staff with respect to clinical intervention and process was capable to guarantee compliance to anticoagulant treatment and distress reduction [45]. 

### 2.1. VTE Current Guidelines 

#### 2.1.1. VTE Prophylaxis

In the light of all the above, it is evident that VTE prevention in cancer patients is of great importance due to the difficult management of thromboembolic events that increase morbidity, interfere with the anticancer treatments causing drug administration delay or discontinuation, affect patient’s QoL and influence disease outcome [46]. 

As regard primary thromboprophylaxis, there is an unanimous consent from all major international guidelines to recommend thromboprophylaxis only in patients hospitalized for acute medical illness and in patients undergoing major surgery, while no thromboprophylaxis is recommended in ambulatory patients receiving chemotherapy due to the limited documented benefits counterbalanced by an increased risk of bleeding [35,47,48,49,50,51,52,53]. Therefore, clinicians are left with the decision to start an anticoagulant prophylaxis in selected high risk categories of patients after careful assessment of the risk of mortality and morbidity associated with possible bleeding events. Although most cancer patients are not recommended to receive prophylactic anticoagulation from VTE, NCCN guidelines suggest prophylactic anticoagulation or aspirin use in patients with multiple myeloma receiving thalidomide, lenalidomide or pomalidomide treatment [47]. The recently updated ASCO guidelines introduced the use of direct oral anticoagulants (DOACs) in VTE prophylaxis and treatment by stating that the use of primary prophylaxis “should” be offered to cancer patients hospitalized for an acute illness or reduced mobility and it “may” be offered to hospitalized solid cancer patients without additional risk factors, provided the risk of bleeding is absent [54]. In a context of critically ill patients such as those in the Intensive Care Units (ICU), anticoagulants should be administered until mobility is restored [55]. All patients with malignant disease undergoing major surgery should be offered thromboprophylaxis with either unfractionated heparin (UFH) or low-molecular weight heparins (LMWHs) unless contraindicated because of active bleeding or high bleeding risk or other contraindications and should be commenced preoperatively [54]. From the above, it is clear the urge to identify those subjects to be treated with antithrombotic prophylaxis. To the purpose of evaluating VTE risk in cancer patients, the Khorana Score (KS), a user-friendly VTE risk predictor based on the evaluation of pre-chemotherapy routinely available variables, has been developed and, despite the acknowledged limitations, has been adopted in the decision making processes (Table 1) [56]. According to KS, patients with a score ≥3 are classified as high-risk, those with a score 1–2 are classified as intermediate-risk and patients with a score = 0 as low-risk ones [56].

Although routine thromboprophylaxis is still not recommended for all cancer outpatients receiving chemotherapy, current guidelines encourage to evaluate patients at higher risk of VTE by means of KS and recommend thromboprophylaxis with LMWH or DOACs, either apixaban or rivaroxaban, to those patients which have been assigned a risk score ≥2 and not actively bleeding or not at high risk of bleeding [54,57,58]. These considerations find the highest application in patients under a simultaneous care therapeutic program.

Two large randomized controlled studies using DOACs for VTE primary prevention and incorporating KS to target intermediate to high-risk patients, evaluated the efficacy and safety of apixaban 2.5 mg twice daily (AVERT) [59] or rivaroxaban 10 mg daily (CASSINI) [57] in ambulatory cancer patients. Both DOACs significantly reduced the risk of VTE in the primary analysis (5.2% on DOACs and 9.3% on placebo; 95% CI, 0.34–0.90; *p* = 0.02) although at the expense of an increased risk of major bleeding (2.0% on DOACs and 1.0% on placebo; 95% CI, 0.80–4.82; *p* = 0.14) and clinically relevant non-major bleeding (4.6% on DOACs and 3.4% on placebo; 95% CI, 0.80–2.27; *p* = 0.26). However, the net benefit of DOACs considering VTE prevention vs. major bleeding, was in favor of an overall risk reduction of 2.8% with DOACs [60].

Other Standard Committees recommend the Khorana risk score as a tool to identify patients with very high risk of VTE [61,62], although acknowledging that the score has insufficient precision in certain settings, such as lung and pancreas cancers [63,64,65].

Other authors have tried to improve the Khorana score performance and proposed modifications by adding biomarkers, types of chemotherapy or performance status [52,66,67]. A recently published review aimed at the optimization of thromboprophylaxis in cancer patients has been considering all these aspects and suggested that prediction scores might be developed for specific cancer sites [68]. Pabinger et al. [69] developed and validated a nomogram that included only tumor site risk category and D-dimer to assess the risk of VTE in chemotherapy-treated cancer patients [69]. Pabinger’s nomogram was validated in an external cohort by Ferroni and co-workers for cumulative 6-month VTE risk prediction. [70]. 

All these models, designed to punctually evaluate ambulatory cancer patients before the starting of a new chemotherapy regimen, do not consider, nor apply, to those admitted to palliative care or hospices. However, they well fit to cancer patients under simultaneous care, thus still under active anticancer treatment. Indeed, among the patients for whom anticoagulation is of uncertain benefit there are listed patient receiving end-of-life/hospice care [54]. Hence, primary thromboprophylaxis for VTE is differently considered in the two setting, palliative and simultaneous (Table 2). The latter might be assimilated to that of the ambulatory cancer patients and evaluated accordingly.

In the dynamic evaluation of patients in simultaneous care, VTE risk assessment might benefit from the inclusion of all these indexes and scores that might combine for the optimization of a unique, inclusive score. As recently outlined by our research group, artificial intelligence (AI) can be used to analyze a huge amount of clinical variables thus representing a solid instrument to build a predictive tool for VTE risk assessment in chemotherapy-treated cancer outpatients [73,74]. This tool has proven extremely useful in selecting VTE risk predictors [73], resulting in a significant improvement of VTE risk prediction performance over the KS [56] and also over the nomogram proposed by Pabinger et al. [69] and can be easily applied to different situations/populations, thus in patients that move from one intensity of care to another, even in the palliative setting.

#### 2.1.2. VTE Treatment and Prevention of Recurrence

Active cancer is a strong risk factor also for VTE recurrence and VTE patients with active cancer should be treated with prolonged anticoagulation therapy as long as the disease is considered active. This, however, poses serious challenging problems due to the increased risk of hemorrhages in this setting of patients, thus a careful evaluation should be performed on a case-by-case basis, since both the differences in the rate of VTE recurrence incidence and major bleeding events are dependent on the cancer type and stage and on associated co-morbidities. Indeed, according to the RIETE study results, cancer patients with VTE recurrence, particularly if the event is a PE, are at a 3-fold increased risk of death [75]. Thus, in cancer patients with established VTE, according to the American Society on clinical Oncology (ASCO) guidelines, initial anticoagulation may involve LMWH, UFH, fondaparinux or rivaroxaban. For long-term anticoagulation, LMWH, edoxaban or rivaroxaban should be used for at least 6 months and preferred to Vitamin K antagonists, which may be used if LMWH or DOACs are not accessible. Further prolongation of anticoagulation for patients with active cancer, should be reserved only to selected patients with metastatic cancer or those receiving chemotherapy [54]. The guidelines released by the Scientific and Standardization Committee (SSC) of the International Society on Thrombosis and Hemostasis (ISTH) [58] suggested the use of LMWHs for cancer patients with an acute diagnosis of VTE and a high risk of bleeding, indicating edoxaban and rivaroxaban as an acceptable alternative if there are no drug–drug interactions with the current systemic therapy. With regard to rivaroxaban, results from the SELECT-D study, showed that it was indeed efficacious in reducing the rate of recurrent VTE compared with LMWH but at the cost of more bleeding, both major and clinically relevant nonmajor bleeding (CRNMB) [76]. A very recent trial (the Caravaggio trial) assessing the efficacy and safety of apixaban during the initial 6-month treatment of venous thromboembolism in patients enrolled without limitation of cancer type and anticancer treatment in order to be consistent with the cancer distribution in the general population, demonstrated a noninferiority of this DOAC (10 mg twice daily for the first 7 days, followed by 5 mg twice daily) as compared to subcutaneous dalteparin, in terms of recurrent VTE (5.6% vs. 7.9%, respectively) and major bleeding (3.8% vs. 4.0%, respectively), including gastrointestinal ones [77].

After 6 months’ treatment, the need for extending anticoagulation requires reassessment in a risk vs. benefit manner, taking into account patient’s preferences [47,71]. The Hokusai VTE Cancer trial, designed to compare, for 6 to 12 months, edoxaban with dalteparin for VTE treatment in patients with predominantly advanced cancer and acute symptomatic or incidental venous thromboembolism, demonstrated a noninferiority of edoxaban with respect to dalteparin in the composite outcome of recurrent venous thromboembolism or major bleeding [78]. Indeed, a post-hoc analysis of the Hokusai-VTE Cancer study patients, demonstrated that an extended treatment (beyond 6 months) with oral edoxaban was as effective and safe as subcutaneous dalteparin [79]. Results from a phase III, multicenter, randomized, double-blind, trial (EVE Trial) assessing apixaban 2.5 mg vs. 5 mg twice daily for 12 months for the secondary VTE prevention in cancer patients who have completed 6 months (but no more than 12 months) of anticoagulation (NCT03080883) are awaited [80].

NCCN guideline recommend lifelong anticoagulation for non-catheter-related cancer DVT or PE while cancer is active, under treatment or if risk factors for recurrence persist [47].

The final treatment strategy should thus be designed by the physician after shared decision-making with the patients, incorporating their preferences and values [58]. In this light, a particular cluster of patients for whom the risk of recurrent VTE and the advantages of oral therapy need to be carefully balanced, is represented by those with gastrointestinal cancer, given their increased risk of bleeding [81].

Patients who have recurrent VTE while on VKA therapy (in the therapeutic range) or on DOACs (dabigatran, rivaroxaban, apixaban or edoxaban) should switch to treatment with LMWH at least temporarily, while in those with VTE recurrence during LMWH, the dose of LMWH should be increased [82].

One important aspect that should be considered and discussed with patients in order to ensure compliance to anticoagulant treatment is the patient’s preference regarding the modality of drug administration. In fact, some patients find tablets more convenient, thus welcoming DOACs, while others accept low-molecular-weight heparin injections as part of their treatment, despite some drawbacks [83].

In spite of the above, the vast majority of the studies performed to assess the best choice/duration of anticoagulant treatment were not directed to the frailest cancer patients, those with poor performance status or a life expectancy lower than 3 months, in which bleeding and recurrent thromboses are increased [84] and that represent a cluster of hospitalized patients that must be considered separately.

### 2.2. Role of Anticoagulants in Simultaneous and Palliative Care

More than 60 years ago, the first randomized trial on thromboprophylaxis demonstrated that adequate oral anticoagulants were able to significantly reduce new symptomatic VTE occurrence and death while containing excessive hemorrhagic side effects in patients undergoing hip fracture surgery [85]. Since then, a number of randomized controlled trials (RCTs), have confirmed that prevention of VTE is feasible and can possibly be considered as the commonest pharmacologically avoidable acute hospital death [86]. RCTs on prevention or treatment of VTE in cancer patients are mainly focused on settings of active oncological therapies and an estimated life expectancy inferior to three months, such as that attributed to subjects on palliative care, was invariable an exclusion criteria for RCTs [87].

The use of anticoagulants and in particular of LMWHs in hospices and SPCUs is controversial. The real effectiveness of full dose anticoagulants in SPCUs is perceived as minimal, since their benefit in terms of VTE-related symptom relief may be outweighed by excessive risk of bleeding in frail cancer patients [88].

Many patients are admitted with a history of VTE and are on stable LMWH at entry. However, subcutaneous administration, often twice a day, is undoubtedly considered as an extraordinary distress for patients who are symptomatic and compromised. No clear data exist on the impact of LMWHs in delaying or relieving VTE-related symptoms, and, although not the primary objective for palliative care, still no data are available on survival prolongation.

In palliative care, anticoagulants are perceived as unnecessary and their use is generally limited. The highly prevalent VTE in cancer patients on palliation is considered more a negligible epiphenomenon of the deteriorated clinical conditions of the near-end-of-life period than a leading cause of premature death or significant contributor of symptom burden.

Results from the HIDDen trial showed that DVT was not associated with reduced survival (*p* = 0.45) and the use of anticoagulants did not reduce DVT incidence (*p* = 0.17). Moreover, DVT was not associated to symptom burden, except from a significant association with limb edema (*p* = 0.009) [44].

Cai et al. performed a Medline, Embase and the Cochrane Library systematic review searching for studies assessing thromboprophylaxis in palliative care. Among a total of 22 original reports, use of thromboprophylaxis ranged between 4% and 53% [89].

More recently, Noble et al. have reviewed patients attending a clinic for CAT and, by using death notification cross-references, selected those dying within 2 years from CAT clinic referral (*n* = 214). Half of them were found to continue LMWH until death and 11% up to 7 days prior death. Even though no VTE-related symptoms were recorded possibly due to the high therapy adherence, a substantial incidence of clinically relevant bleeding was notified (7%) [90].

In the above-mentioned study by Soto-Cárdenas et al. [42], after VTE diagnosis all patients received LMWH. Consistent hemorrhagic complications were reported (11.3%) and some patients died because of the bleeding (4%). However, in a relevant percentage of cases the death was considered to be VTE-related instead, despite the start of full dose LMWH. Authors concluded that the risk/benefit ratio in this specific cancer population need to be attentively evaluated.

Similar incidence of bleeding complication has been recorded in a French cohort of palliative care cancer patients (*n* = 1091). Overall, bleeding occurred in 10% of patients and in the majority of cases was associated with LMWH. Pharmacological thromboprophylaxis was associated with a nearly 50% increased risk of hemorrhagic event in this patient population (HR: 1.48, *p* = 0.04) [91].

For patients on active anti-cancer treatment, NCCN guidelines recommend indefinite anticoagulation when CAT is diagnosed [47]. However, the real clinical impact of such an approach seems to be of limited value in the palliation setting mainly because of the very short life expectancy. In the above-mentioned HIDDen trial, almost two third (61%) of screened patients did not meet the inclusion criteria because of a believed life expectancy <5 days [44].

These evidences, taken together with the substantial bleeding risk of anticoagulants, suggest that treatment should be started/continued in highly selected cases when a worsening in symptom burden is feared. Moreover, they highlight the need for robust clinical study in the palliative care population to assess the best strategy for VTE prevention and treatment and the most accurate measurable end points relevant to the advanced cancer population [92].

The necessity of VTE risk assessment tools and thromboprophylaxis for patients admitted to palliative care units in order to identify those at higher risk, led to the development of guidelines especially focused on patients admitted to either acute or palliative care settings—the Pan Birmingham Cancer Network (PBCN) palliative-modified Thromboembolic Risk Factors (THRIFT) Consensus Group criteria [93]. The PBCNP Guideline for VTE primary prophylaxis suggests that all patients, regardless of diagnosis, should be assessed through a three-step process involving—1) general assessment; 2) assessment of the benefits of prophylaxis; 3) palliative team decision. The last step, to be also discussed with the patient, considers the appropriateness of treatment weighing up not only the associated risks and benefits but also the burden of monitoring and allows designing a strategy of therapy choice, duration and monitoring.

### 2.3. The Integrated Model

An integrated system of multilevel networks is the optimal way to guarantee the patient access to palliative care and pain therapy. The “network,” as such, is designed to promote patient care continuity, from hospital to home and coordinates the structures and professionals dedicated to providing the service in a context of simultaneous care. Simultaneous care represents the new paradigm of care for cancer patients and requires a cultural and organizational change necessary to share goals, values and programming at the level of operating units, multidisciplinary groups, oncology departments and territorial services. This modality of management and treatment of advanced disease is aimed at associating, in a systematic way, palliative care with anticancer therapies, obtaining not only a benefit on QoL parameters but in some cases, even an extension of survival.

It has become evident that the problems and needs of patients affected by advanced neoplasia and their family members start long before the end of life phase, so that simultaneous care can be considered as the set of global care interventions aimed at both the patients and to family members and, more generally, to caregivers. This concept was implemented in 2012 by ASCO that recommended considering the combination of standard cancer care and palliative care early in the course of the disease, for all patients with metastatic cancer and/or with symptomatic disease [13]. Indeed, an increasing number of patients are admitted to palliative care units or residential hospices for brief periods necessary for symptom assessment and management and are then subsequently discharged home to continue active anticancer treatments, with discharge rates of about 60% [94,95,96].

The integrated management model should be considered the most suitable approach to improve care for people with oncological pathologies, whose effective treatments are necessarily modulated on the different levels of severity. Due to the complexity of the neoplastic pathology, a close collaboration between the many specialists is required in the form of multidisciplinary meetings between specialists from different disciplines. It is, therefore, necessary to identify integrated care and organizational pathways and to use validated multi-dimensional tests for patients in the metastatic phase, to detect and respond to all symptoms and care needs.

According to our own experience, in order to guarantee users a coordinated information flow and a single access to home services, it is essential that the palliative care network, with regard to its home activities, be coordinated and closely connected with a reference Operating Center for the Services of home care. We have, thus, stipulated local operating protocols, agreed upon and predefined between the Central and the Dispensing Subjects constituting the network, which safeguard the patient’s freedom of choice. These protocols will have to consider all the phases of the specific care process for the end of life (reporting, evaluation, acceptance and definition of the care plan, verification of the results), which must be carried out jointly by the general practitioner, the staff of the PCU and the Operating Center of the Home Care Service. Furthermore, for patients included in a home-based palliative care program we have considered appropriate to provide for the direct delivery of drugs (in particular analgesic drugs including opioids and anticoagulants). The cooperative process that ascertains the need, plans, implements, coordinates, controls and evaluates options and services in response to an individual’s demand in order to achieve quality and economically efficient outcomes is defined as Case Management. The Case Management model in Oncology in general and in our Unit in particular, is configured as a highly innovative project having been applied for the first time to sections of the population classified as “fragile,” that is, with great difficulty in accessing and autonomously following medical care and access to the hospital. The model is outlined as a new tool in the course of treating the disease; the person and his centrality have been placed at the base of the realization of the program—“there is no cure for the disease without personal care.”

Patients with medium/low intensity of care (defined as “no-therapy” with hospice transfer/home assistance) are those with advanced cancer, for which health interventions are no longer capable to provide satisfactory results in terms of medium-long term regression-stabilization of neoplastic disease.

Indeed, when cancer patients reach a limited life expectancy, quality of life and symptom relief often represent more important endpoints than survival. These outcome measures are subjective and not always reported [97]. Considering that the vast majority of patients admitted to hospices are at moderate to high risk of developing a VTE during their stay [93], the possibility of an early integration of VTE preventive strategies, in a simultaneous care program, might help overcoming the problem of deciding in favor or against thromboprophylaxis in a context of palliation.

In 2006, the Food and Drug Administration (FDA) introduced the concept of patient-reported outcomes measures (PROMs) for those measures that best reflect the patients’ perceptions, for an optimal monitoring of symptoms from the primary cancer diagnosis and during follow-up care [98]. Advances in information technologies permits to collect PROMs by means of electronic tools, namely electronic PRO (ePRO). The development of such tools allowing the integration of PROMs with patient-related data from hospital and laboratory sources warrants a follow-up at several levels, also at distance, with data that can be automatically transferred in real time to a computer server [99,100,101,102]. Moreover, all clinically relevant actions based on PRO scores can be added to the patient’s electronic medical record, thus allowing the health-care providers to be always aware of patient conditions and to move smoothly from an active treatment to palliation.

Novel tools allowing routine assessment of PROs via smartphone and tablet applications have proven user friendly to patients, with a low loss of data and with the possibility to monitor patient compliance to pharmacotherapy [102,103].

## 3. Physicians’ Perspectives

Specific guidelines for management of VTE in palliative care patients are lacking and administration of anticoagulants relies mainly on physicians’ clinical judgement. Expertise and individual clinical judgment is pivotal in the decision-making process, however it might be more influenced by incidental factors and personal convictions than by objective evidence.

In general, palliative care physicians are less prone to prescribing anti-coagulants and the perceived imminent death for most of cancer patients on palliation is considered the main reason, thus impeding the appropriate prevention of VTE-related symptoms in some cases. On the other hand, in certain sets of cancer patients, such as pancreatic ones, an early VTE episode at the beginning of chemotherapy administered for palliation, represents a poor prognostic factor [104].

In a factorial survey conducted in Canada among 62 medical oncologists (MOs) and 73 palliative care physicians (PCPs), MOs were twice more likely to prescribe anti-coagulants in specific VTE risk conditions (OR: 2.09, *p* < 0.001) [105]. In the multivariable analysis, being a medical oncologist was an independent factor associated with anticoagulant prescription, together with medical conditions that indicate a possibly longer overall survival (such acute care hospital admission or reversible cause for admission) and low risk of bleeding.

PCPs have culturally and historically less attitude towards intensive interventions, however specific differences in the training programme between MOs and PCPs might contribute to different medical decisions in the same clinical scenarios and specific guidelines are eagerly needed to harmonize the standard treatment in this context.

Similar results were found in a smaller study surveying a diverse panel of 20 physicians constituted of experts in palliative care, oncology, blood coagulation and intensive care. Again, PCPs were less likely to indicate thromboprophylaxis.

**Figure 1:**
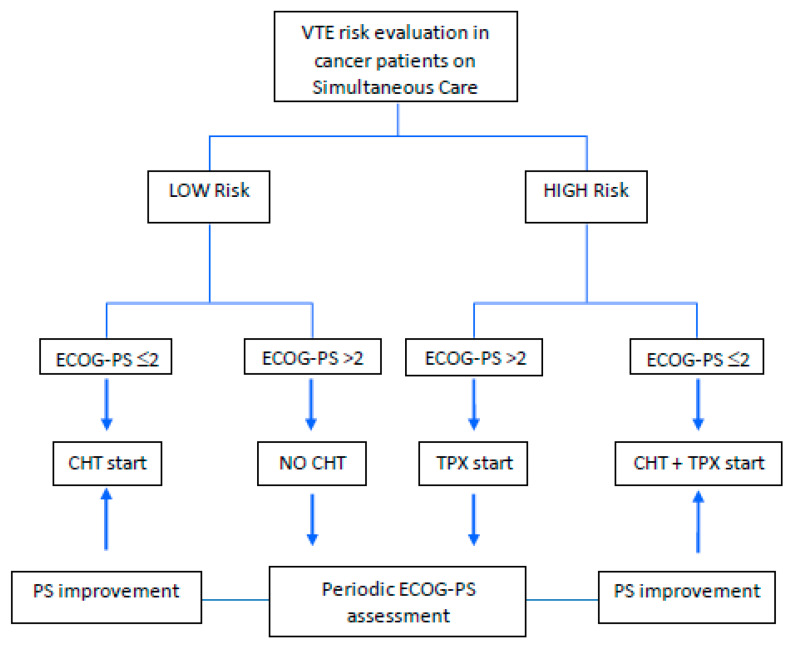
Proposed algorithm for a therapeutic strategy based on the evaluation of venous thromboembolism (VTE) risk in patients with advanced cancer on a simultaneous care program. The classical category of the Intermediate-risk patients defined by the Khorana score is no longer included, since the integration of detailed programs and evaluations allows a more specific discrimination among patients [68,69,70,73]. CHT: Chemotherapy; ECOG-PS: ECOG-Performance Status; TPX: Thromboprophylaxis.

This possibly nihilistic approach among PCPs (VTE perhaps conceived as one of the possible terminal causes of an imminent death) is in contrast with the increasing percentage of patients being discharged from the palliative care settings because admitted and treated for reversible causes for brief periods [106]. Conversely, larger consensus might come from the inclusion of patients in the simultaneous care setting under the category of ambulatory cancer patients, for which a constant evaluation of pros and cons should indicate the appropriate timing and risks of anticoagulation. Simultaneous cares are indeed increasingly involved in the earlier phases of the cancer journey when active anticancer treatments are still delivered and intensive interventions are required [95]. Specific decision-making tools are necessary to avoid under-treatment also in the field of CAT and since the continuum of care paradigm is in constant change, a major effort should be made in this area to achieve a broad consensus on how to manage VTE. Figure 1 depicts a proposed algorithm for the management of the best anticancer strategy to cancer patients under a simultaneous care program that includes palliative care interventions with active antineoplastic treatment and cannot ignore an initial VTE risk evaluation. 

## 4. Conclusions

People are living longer thus the aging population associated with increased multimorbidity, chronic diseases and disability is growing and is living longer with metastatic disease for which it is receiving more and more chemotherapy and palliative therapies.

In palliative clinical practice, oncologists are frequently faced with the task of determining the appropriate, if any, anticoagulation strategy in their patients. The first crossroads is represented by the necessity to establish whether risk of VTE occurrence overcomes the risk of fatal bleeding. Secondly, the possibility to switch the patient to active anticancer treatment during his/her staying should be determined. Indeed, anticoagulant therapy may have important side effects that could cause cancer treatment discontinuation and could even result in patient’s death. Finally, when patients are diagnosed with VTE, the period in which VTE occurred, either before or during admission, could guide the decisions to start anticoagulation. Information regarding prognostic VTE-related factors and predictors would assist oncologists in predicting the occurrence of VTE and in determining active cancer treatment as well as anticoagulant therapy in clinical practice. On the other hand routine risk assessment for VTE in all patients admitted to a hospice is not usual and hospices are managing patients who are not imminently dying. Thus, it is important a careful evaluation of the effects of VTE and its related events on QoL and, conversely, those of anticoagulant treatment. In this light, the possibility to realize algorithms that include patient’s age, co-morbidities and polypharmacy, might enhance the sensitivity of existing available biomarkers and might allow the discovery of new, more specific, ones along with the development of appropriate testing for this particular cluster of patients. This will represent a fundamental step to avoid delays of VTE thromboprophylaxis and to allow an early start during the course of the active cancer treatment, according to a simultaneous care model.

## Figures and Tables

**Table 1:** The Khorana risk assessment model for cancer patients prior to chemotherapy start [56].

Patient’s Characteristics	Score
Site of cancer Very high risk (stomach, pancreas, brain)	2
High risk (lung, lymphoma, gynecologic, bladder, myeloma, testicular or kidney)	1
Platelet count ≥350 × 10^9^/L	1
Hemoglobin level <6.2 mmol/L or use of red cell growth factors	1
Leukocyte count >11 × 10^9^/L	1
Body mass index ≥35 kg/m^2^	1

The total score represents three risk groups of patients: 0 = low risk, 1–2 = intermediate risk, 3 = high risk.

**Table 2:** Recommendation guidelines for thromboprophylaxis in cancer patients. Differences between palliative care and ambulatory (in which patients on simultaneous care can be reconsidered) settings.

Guideline	Recommendation	
	Palliative Care	Ambulatory Setting
National Institute for Health and Clinical Excellence (NICE) [71]	TP should be considered for hospitalised palliative care patients, taking into account temporary increases in thrombotic risk factors, risk of bleeding, likely life expectancy and the views of patient and caregivers. Exceptions are patients in the last days of life.	Not specifically addressed. TPX is not indicated in patients receiving cancer-modifying treatments such as RT, CHT or immunotherapy, unless they are also at increased risk of VTE for other reasons than cancer. Consider for people receiving CHT for pancreatic cancer or myeloma (in association with thalidomide, pomalidomide or lenalidomide and steroids).
American College ofChest Physicians (ACCP) [51]	No guidelines in palliative care. Recommended in immobilized outpatients with solid tumors but opposed in immobilized patients at nursing homes.	TPX is not recommended routinely but it is suggested in those patients with additional risk factors for VTE and who are at low risk of bleeding
British Committee forstandards in Haematology (BCSH) [62]	Antithrombotic use aimed solely at increasing life expectancy in patients with cancer but without a history of VTE, is not recommended	Outpatients with active cancer should be assessed for thrombosis risk; TPX should be considered for high risk patients and offered to patients with myeloma receiving thalidomide or lenalidomide, unless contraindicated
National Comprehensive Cancer Network (NCCN) [47]	No guidelines in palliative care. Routine TPX use should be limited to clinical trials only	Patients with a KS score ≥3 could be considered for VTE prophylaxis on an individual basis, after discussions with patients/caregivers regarding the potential risks and benefits. Prophylactic anticoagulation or aspirin use in patients with multiple myeloma receiving thalidomide, lenalidomide or pomalidomide treatment, is suggested
American Society of Clinical Oncology (ASCO) [54]	No guidelines in palliative care. TPX should not be the life-prolonging procedure. Can be considered in selected high-risk cancer outpatients	Routine TPX should not be offered. In high-risk outpatients (KS≥ 2) it may be offered provided there are no significant risk factors for bleeding nor drug interactions. Patients with multiple myeloma receiving thalidomide- or lenalidomide-based regimens with chemotherapy and/or dexamethasone should be offered pharmacologic thromboprophylaxis with either aspirin or LMWH for lower-risk pts and LMWH for higher-risk pts
European Society for Medical Oncology (ESMO) [52]	No guidelines in palliative care setting	Routine TPX is not recommended apart from select populations of cancer patients with solid tumours or in categories of patients with myeloma.
International Society on Thrombosis and Haemostasis (ISTH) [60]	No guidelines in palliative care setting	Primary TPX is suggested in cancer patients starting chemotherapy with a KS ≥2, no drug-drug interactions and not at high risk for bleeding
International Initiative on Thrombosis and Cancer (ITAC) [61]	No guidelines in palliative care setting. TPX is suggested in hospitalised patients with reduced mobility	Primary prophylaxis is not recommended routinely but indicated in patients with locally advanced or metastatic pancreatic cancer treated with systemic anticancer therapy and who have a low risk of bleeding
Italian Association of Medical Oncology AIOM [72]	No guidelines in palliative care setting	TPX is not routinely recommended in patients at low risk but it can be considered only in high risk patients receiving chemo- or hormone-therapy.
Canadian Consensus Recommendations [32]	No guidelines in palliative care setting. Hospitalized patients with active malignancy and acute illness or decreased mobility should receive TPX in the absence of contraindications.	TPX is not routinely recommended. May be considered for very selected high-risk patients receiving chemotherapy.

CHT: chemotherapy; KS: Khorana Score; RT: Radiotherapy; TPX: Thromboprophylaxis; VTE: Venous thromboembolism.
